# Supplementary material for: In vivo characterization of a retinal cellular biomarker of inflammation in multiple sclerosis
Source: Brain Commun. 2025 Nov 28;8(1):fcaf471. doi: 10.1093/braincomms/fcaf471 (PMC12957951; doi:10.1093/braincomms/fcaf471)
Supplement: fcaf471_Supplementary_Data [file fcaf471_Supplementary_Data.pdf]

Supplementary Figures and Tables

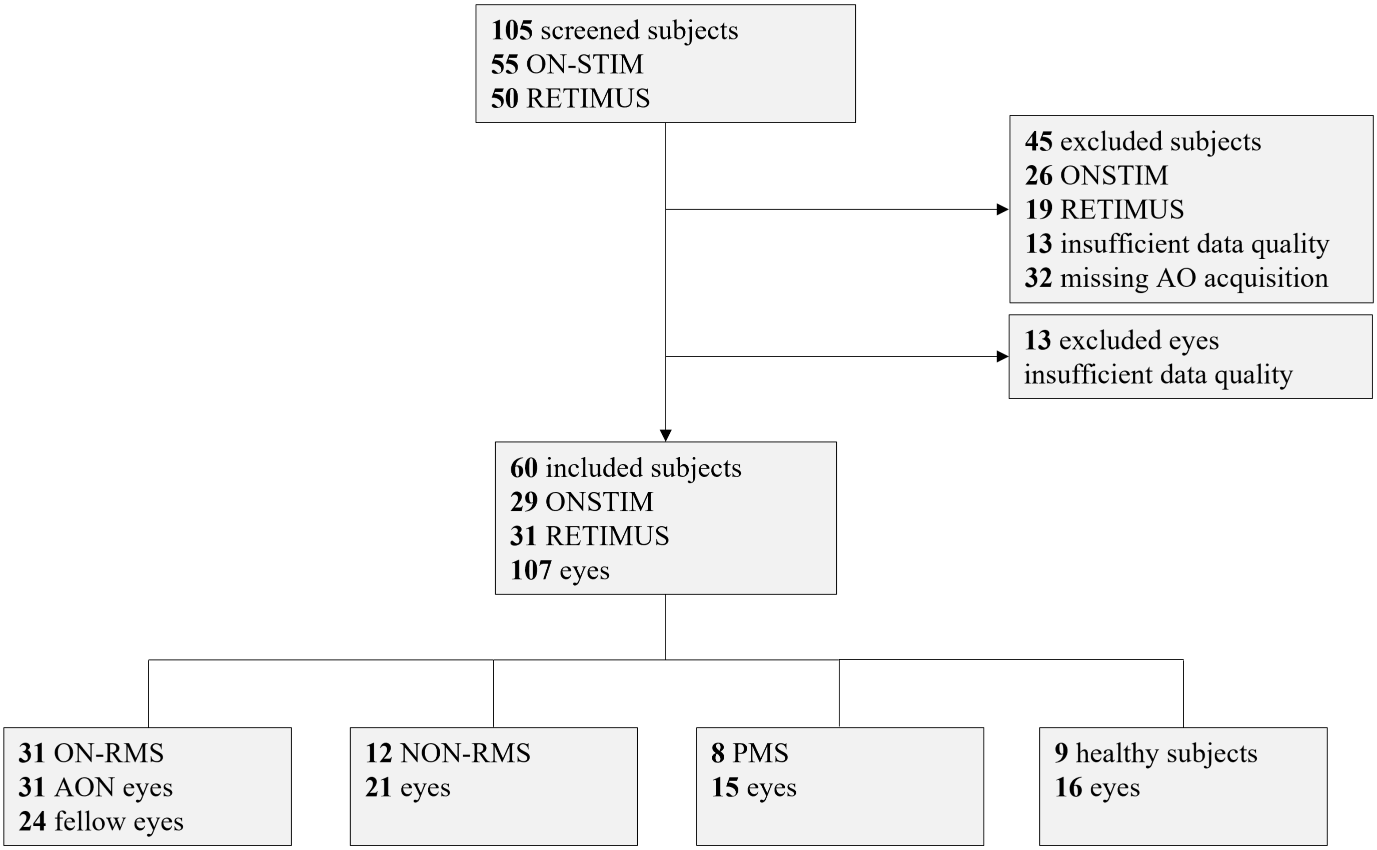

Supplementary Figure 1. Flow chart illustrating patient inclusion from two clinical studies, ON-STIM (ClinicalTrials.gov ID: NCT04042363) and RETIMUS (ClinicalTrials.gov ID: NCT04289909), along with reasons for data exclusion. Exclusions include missing AO data due to system upgrades or the absence of a trained imaging technician, as well as poor data quality caused by fixation issues or excessively thick ocular media. The figure also shows the distribution into the four groups of patients with MS and controls. AO: Adaptive Optics; ON-RMS: Relapsing-Remitting Multiple Sclerosis with recent acute optic neuritis; NON-RMS: Relapsing Multiple Sclerosis without recent acute optic neuritis; PMS: Progressive Multiple Sclerosis.

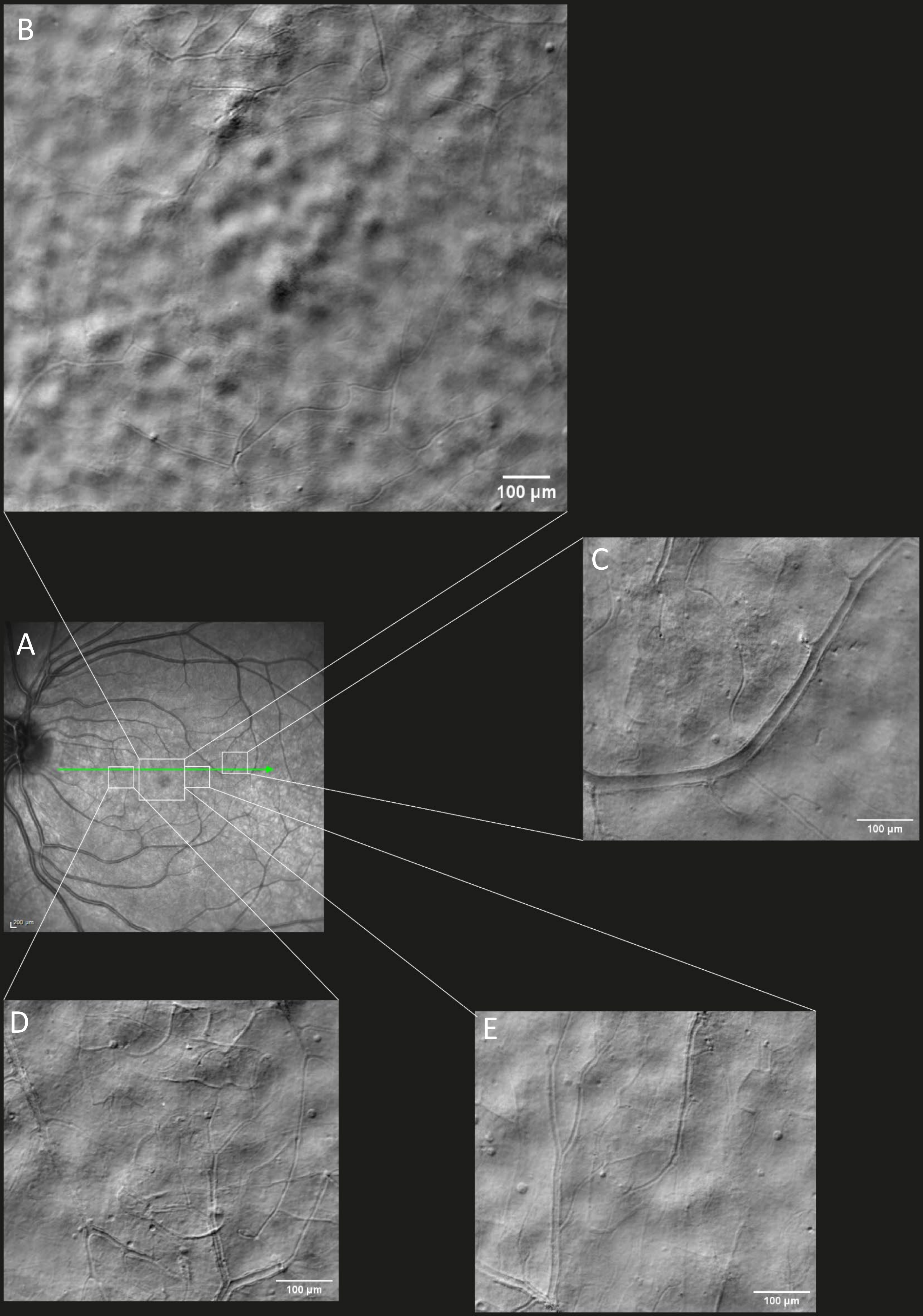

Supplementary Figure 2. (A) Scanning Laser Ophthalmoscope (SLO) image in an ON-RMS (Relapsing-Remitting Multiple Sclerosis with recent optic neuritis) patient. (B-E) Off-axis AOSLO (Adaptive Optics Scanning Laser Ophthalmoscope) images at retinal eccentricities beyond the study 4° temporal and nasal imaging protocol revealing immune cells in additional retinal regions.

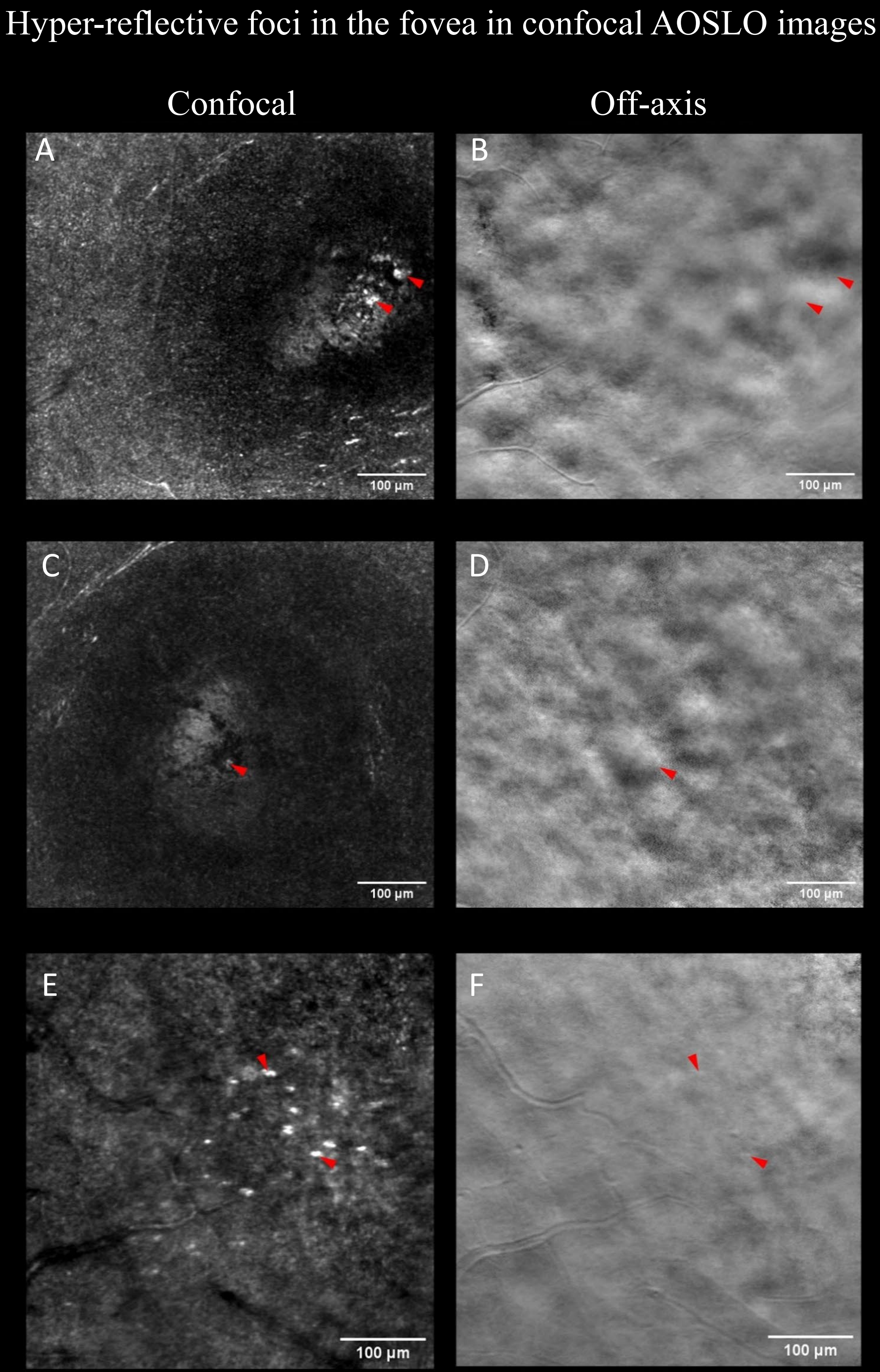

Supplementary Figure 3. (A,C,E) Confocal AOSLO (Adaptive Optics Scanning Laser Ophthalmoscope) images of the fovea from three multiple sclerosis patients: (A) one ON-RMS, (C,E) two NON-RMS showing hyperreflective dots previously described by Hargrave et al [1]. These structures do not produce a phase contrast signal in off-axis AOSLO images (B,D,F), indicating they are distinct from the cells described in this study. Red arrows indicate hyperreflective dots in confocal images, which are absent in off-axis images.

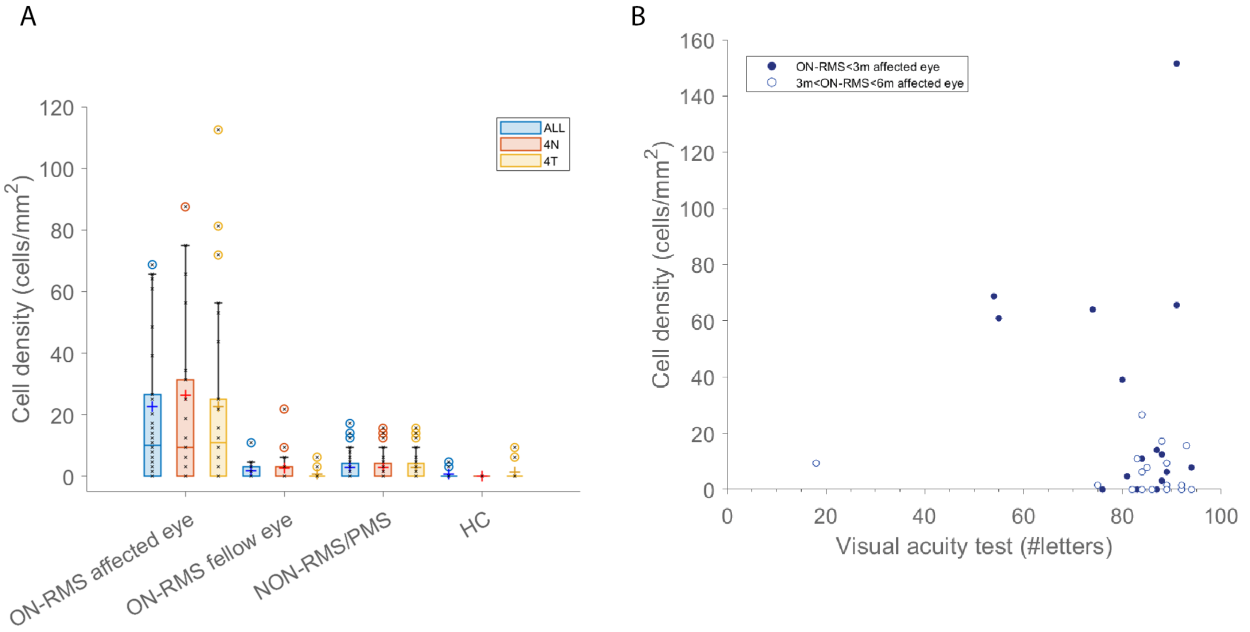

Supplementary Figure 4. (A) Influence of nasal vs. temporal retinal location (4° Nasal (4N), 4° Temporal (4T) and both 4T and 4N (ALL)) on cell density at baseline (first AOSLO visit) across multiple sclerosis (MS) subtypes (ON-RMS: Relapsing MS with recent optic neuritis affected eye (N=31), ON-RMS fellow eye (N=24), NON-RMS: Relapsing MS without recent Optic Neuritis/PMS: Progressive MS (N=36)) compared to Healthy Controls (HC, N=11). (B) Influence of visual acuity (low contrast) on cell density at baseline in patients from ON-RMS group. Each data point represents a cell density measurement from the affected eye of an ON-RRMS patient at baseline, plotted against visual acuity results (N=20). No significant correlations were observed between eccentricities (4N, 4T, ALL) in either group, as assessed by non-parametric Spearman correlation (p>0.05).

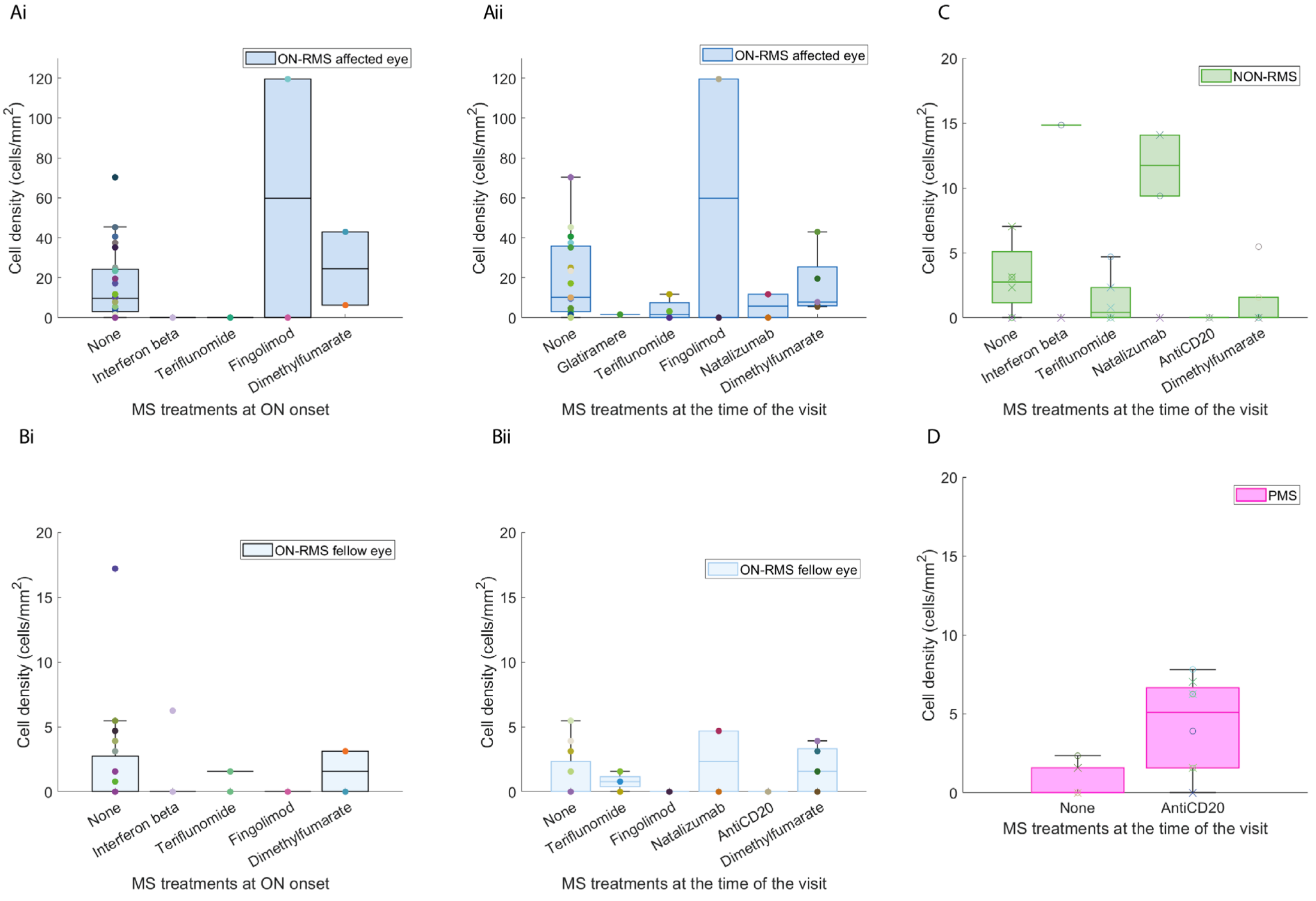

Supplementary Figure 5. Cell density at first AOSLO (Adaptive Optics Scanning Laser Ophthalmoscope) visit (baseline) plotted against disease-modifying treatments in multiple sclerosis (MS). (Ai, Bi) Cell density in the affected (N=31) and fellow (N=24) eyes of ON-RMS (Relapsing-Remitting MS with optic neuritis) patients, respectively, for different MS treatments at ON (optic neuritis) onset. (Aii, Bii, C, D) Cell density for different treatments at the time of the visit for ON-RMS affected (N=31) and fellow eyes (N=24), as well as for NON-RMS (RMS without optic neuritis, N=21) and PMS (progressive MS, N=15) patients, respectively. Non-parametric Spearman correlation was computed and we did not find any association between retinal cellular density and MS treatments (p>0.05).

Supplementary Table 1. Cells mean and median diameter for all MS groups and healthy controls

Group	Mean size (μm)	Median size (μm)	Standard Deviation (μm)
**ON-RMS affected eye**	12.8	12.4	3.5
**ON-RMS fellow eye**	14.9	13.8	6
**NON-RMS**	11.1	11.1	2.7
**PMS**	10.3	10.7	1.7
**HC**	10.9	10.5	2.9

Acronyms: ON: Optic Neuritis; ON-RMS: Relapsing Remitting MS patients with recent optic neuritis; NON-RMS: Relapsing Remitting MS patients without recent optic neuritis; PMS: Progressive Multiple Sclerosis patients; HC: Healthy Subjects.

References

[1] Hargrave A, Sredar N, Razeen MM, et al. Novel microscopic foveal pit pathology in multiple sclerosis revealed with adaptive optics ophthalmoscopy. *Investigative Ophthalmology & Visual Science*. 2020;61(7):5101-5101..
